# High-Throughput Sequencing Survey of Sunflower Soil

**DOI:** 10.1128/MRA.01331-20

**Published:** 2021-02-25

**Authors:** Olubukola Oluranti Babalola, Blessing Chidinma Nwachukwu, Ayansina Segun Ayangbenro

**Affiliations:** a Food Security and Safety Niche, Faculty of Natural and Agricultural Sciences, North-West University, Mmabatho, South Africa; Indiana University, Bloomington

## Abstract

Plant rhizobiomes are responsible for major soil processes in the immediate plant environment, but our knowledge of the linkage between below-ground microbiota diversity and plant health is limited. We studied the bacterial and archaeal communities of sunflower rhizosphere organisms by comparing the composition of these communities to bulk soils at three farms in the North West province of South Africa. We evaluated and described a plethora of bacterial and archaeal taxa.

## Abstract (precis)

Plant rhizobiomes are responsible for major soil processes in the immediate plant environment, but our knowledge of the linkage between below-ground microbiota diversity and plant health is limited. We studied the bacterial and archaeal communities of sunflower rhizosphere organisms by comparing the composition of these communities in bulk soil at three farms in the North West province of South Africa. We evaluated and described a plethora of bacterial and archaeal taxa.

## ANNOUNCEMENT

South Africa, as one of the semiarid areas in the world, is a gold mine of important soil microorganisms with various functions that could be used for biofertilization. Sheila, Itsoseng, and Kraaipan are towns located in the Ngaka Modiri Molema local district municipality (North West Province). North West Province, after Free State, is the bedrock of sustainable sunflower production, accounting for around 80% of sunflower plantations and contributing significantly to the economy (1). The high productivity of the study areas can be attributed to the plant growth-promoting rhizobacteria present in the soil. This study was designed to determine the structure of sunflower rhizospheric soil bacterial and archaeal communities of the study areas.

Soil samples were obtained from sunflower rhizosphere and bulk soils from three farms in North West Province. Farm soil samples came from Sheila (SH) (26°2′41.202″S,25°57′47.49″E), Itsoseng (IT) (26°4′23.064″S, 25°58′37.104″E), and Kraaipan (KP) (26°17′24.186″S, 25°13′33.258″E). The rhizosphere soils were collected using a destructive sampling method in which sunflower plants were uprooted from an area of 2 by 4 m^2^. Then, the soil that was attached to the plant roots after shaking the uprooted plant was collected and kept in a sterile plastic bag. The bulk soils were collected 10 m from each site where sunflower rhizosphere soils were collected and also kept in sterile plastic bags (2). The soil samples were immediately transported in an ice-packed container to the laboratory and stored in a cold room at 4°C prior to analysis. A kit (Zymo, California, USA) was used to extract microbial DNA from 2 g of each soil sample according to the manufacturer’s instructions.

Sample sequencing was performed using an Illumina MiSeq instrument at Molecular Research LP (Shallowater, TX, USA). The purity and concentration of the DNA samples were analyzed using NanoDrop ND-2000 and Qubit DNA broad-range (BR) reagent assays. 16S rRNA libraries were obtained using the quality control (QC)-passed DNA samples with the PCR primers 515F (5′-AATGATACGGCGACCACCACCGAGATCTACAC TATGGTAATTGTGTGCCAGCMGCCGCGGTAA-3′) and 806R (5′-CAAGCAGAAGACGGCATACGAGATTCCCTTGTCTCCAGTCAGTCAGCCGGACTACHVGGGTWTCTAAT-3′) (3) with adapters and standard Illumina barcodes. The DNA samples were purified using AMPure XP beads. As a quality control measurement, the barcoded libraries were validated using the Agilent DNA 1000 bioanalyzer, and the concentration was quantified using a Qubit assay kit. The libraries were merged and sequenced on the Illumina MiSeq platform. The raw sequences were processed and analyzed on MG-RAST server v4.0.3 (http://metagenomics.anl.gov/) (4). After demultiplexing of the paired-end reads, the raw data were uploaded as FASTQ files. Quality processing and deduplication by MG-RAST pipeline analysis generated reads which were subjected to taxonomic analysis (4). Then, the bacterial and archaeal abundances present in the Sheila, Itsoseng, and Kraaipan soils were estimated (4). All bioinformatics tools were run with default parameters (5).

At the domain level, the taxonomic descriptions of archaea and bacteria showed 7.0 to 13.4% and 86.59 to 99.30% mean read values, respectively. Bacterial phyla such as *Firmicutes* (17 to 51%), *Proteobacteria* (18 to 36%), and *Actinobacteria* (7 to 38%) were the most abundant. Archaeal reads were allotted to *Thaumarchaeota* (1 to 13%) and *Crenarchaeota* (1%) abundance. The numbers of raw sequence reads of each sample, which are higher than the corresponding numbers of reads that passed quality control (QC), are shown in Table 1. Quality control of the metagenomics data was done to remove low-sequencing reads and contaminating reads (e.g., reads of eukaryotic species). Since this study is based on the 16S amplicon, the number of reads that passed QC is lower than the number of raw reads, possibly because the samples contained more eukaryotic species, such as fungi, that were identified and removed during the quality control step.

**TABLE 1:** Sequence information from MG-RAST and NCBI databases for the analyzed soil samples

Sample	Description	NCBI BioProject no.	SRA accession no.	No. of raw sequence reads	No. of reads that passed quality control	Mean sequence length (bp)
S1_A	SH rhizosphere soil replicate 1	PRJNA672856	SRR12960272	138,303	17,428	287 ± 17
S2_B	SH rhizosphere soil replicate 2	PRJNA672856	SRR12960271	112,226	11,684	288 ± 13
S3_C	SH bulk soil replicate 1	PRJNA672856	SRR12960268	116,529	12,217	290 ± 14
S4_D	SH bulk soil replicate 2	PRJNA672856	SRR12960267	57,330	8,511	268 ± 66
S5_M	IT rhizosphere soil replicate 1	PRJNA672856	SRR12960266	101,263	14,962	291 ± 10
S6_N	IT rhizosphere soil replicate 2	PRJNA672856	SRR12960265	82,616	14,313	291 ± 8
S7_O	IT bulk soil replicate 1	PRJNA672856	SRR12960264	81,580	14,921	291 ± 10
S8_P	IT bulk soil replicate 2	PRJNA672856	SRR12960263	95,189	16,961	291 ± 10
S9_Q	KP rhizosphere soil replicate 1	PRJNA672856	SRR12960262	123,996	17,947	287 ± 15
S10_R	KP rhizosphere soil replicate 2	PRJNA672856	SRR12960261	148,667	11,014	287 ± 19
S11_S	KP bulk soil replicate 1	PRJNA672856	SRR12960270	115,727	15,742	288 ± 14
S12_T	KP bulk soil replicate 2	PRJNA672856	SRR12960269	138,303	17,428	287 ± 17

### Data availability.

The raw sequence files (reads in FASTQ format) were deposited at the NCBI SRA database under the BioProject number PRJNA672856 for samples SH (samples S1_A, S2_B, S3_C, and S4_D), IT (samples S5_M, S6_N, S7_O, and S8_P), and KP (samples S9_Q, S10_R, S11_S, and S12_T). The samples can be accessed under SRA accession numbers SRR12960272, SRR12960271, SRR12960268, SRR12960267, SRR12960266, SRR12960265, SRR12960264, SRR12960263, SRR12960262, SRR12960261, SRR12960270, and SRR12960269. The quality-filtered and annotated data for individual replicates have been released publicly in the MG-RAST database with the accession numbers mgs831279 (sample S1_A), mgs831282 (sample S2_B), mgs831285 (sample S3_C), mgs831300 (sample S4_D), mgs831303 (sample S5_M), mgs831306 (sample S6_N), mgs831309 (sample S7_O), mgs831312 (sample S8_P), mgs831270 (sample S9_Q), mgs831279 (sample S10_R), mgs831273 (sample S11_S), and mgs831276 (sample S12_T).
